# Leveraging Game-Based Learning Technologies to Introduce Adolescents to Health Science Careers During the COVID-19 Pandemic

**DOI:** 10.15695/jstem/v4i4.09

**Published:** 2021-10-04

**Authors:** Randall Spain, Carlos Penilla, Elizabeth Ozer, Robert Taylor, Cathy Ringstaff, James Lester

**Affiliations:** 1 Center for Educational Informatics, North Carolina State University, Raleigh, NC; 2 Division of Adolescent and Young Adult Medicine, Department of Pediatrics, University of California, San Francisco, CA; 3 Office of Diversity & Outreach, University of California, San Francisco, CA; 4 WestEd, San Francisco, CA

**Keywords:** Game-based learning, health science careers, COVID-19

## Abstract

The COVID-19 pandemic produced a dramatic nationwide shift in K-12 education from in-person classroom learning to remote online learning. This shift left teachers and parents facing the challenge of finding engaging online resources to motivate students to become deeply involved in science learning. The pandemic also left educators and researchers, whose work focuses on providing students with experiential learning opportunities in the sciences, with the challenge of adapting to virtual and remote models to continue engaging students in STEM learning activities. In this article we describe: 1) the Health Quest project, which centers on the development of technology-rich learning resources to promote middle grade students’ interest in health science careers, with a focus on girls and underrepresented racial and ethnic minorities; and 2) how the project has responded to the challenges presented by the COVID-19 pandemic. In Health Quest, through engaging narrative-based learning scenarios, students work with virtual characters to experience health science careers from multiple perspectives. Although originally envisioned for in-person classroom learning, we discuss how the team is adapting the Health Quest Career Adventure Game to remote learning, including highlighting the role science plays in addressing public health outbreaks. We describe new gameplay features that have been added to support career modeling and how we have adapted the core technology underpinning Health Quest to support broad dissemination to meet the project’s broader goal of increasing adolescents’ interest in and self-efficacy for pursuing health science careers. We conclude with a discussion of how our evaluation strategies have changed from in-person focus groups and testing to an online data collection model and lessons learned.

## INTRODUCTION

The COVID-19 pandemic produced a dramatic change in K-12 classrooms across the nation. Students and teachers shifted from learning and teaching in physical classrooms where they can work and engage in-person to online classrooms where students engage with digital learning content and hold class meetings and discussions remotely over video conferencing. This shift left educators as well as parents with the challenge of finding compelling virtual learning content to engage students in science learning. The pandemic also impacted educators and researchers who support in-person programs that aim to provide students with experiential learning opportunities in the sciences with the challenge of adapting to virtual and remote learning models to continue to engage students in Science, Technology, Engineering and Mathematics (STEM) learning activities. While optimistic that in-person classroom learning will resume this academic year, the lessons learned in these shifts to virtual learning environments will continue to inform K-12 teaching.

Game-based learning environments hold significant promise for addressing the challenges caused by the COVID-19 pandemic by engaging students in deeply immersive learning experiences that can increase student motivation and promote learning through interactive gameplay (Plass et al., 2020). A growing body of work on game-based learning environments that incorporate pedagogical principles such as scaffolding, feedback, and guided learning experiences can yield positive learning outcomes across a range of subjects and settings (Connolly et al., 2012; Lester et al., 2020; Martinez-Garza et al., 2013; McClarty et al., 2012). Meta-analyses have also shown that game-based learning can be more effective with respect to learning and retention than traditional instructional methods (Clark et al., 2016; Wouters et al., 2013).

In this article, we describe the Health Quest project, which centers on the development of game-based learning resources to promote adolescents’ interest in health science careers, and how the team has responded to the challenges presented by the COVID-19 pandemic. In particular, we discuss how the research team has adapted the interactive game-based resources to highlight the role science plays in addressing public health outbreaks. We also discuss how we have adapted the core technology underpinning Health Quest to support broad dissemination to meet the project’s goal of increasing adolescents’ knowledge of and interest in pursuing health science careers, and how we have adapted our evaluation strategies from in-person focus groups and testing to a remote data collection model. We conclude with a discussion of lessons learned and the research team’s implementation plan moving forward.

### Background and Motivation of Health Quest.

Developing a diverse health sciences workforce is a critical national need. Significant workforce shortages exist and are emerging in the fields of medicine (Zhang et al., 2020), dentistry (Wood and Gaid, 2018), public health (Bogaert et al., 2019), nutrition/dietetics (Delisle et al., 2017), and behavioral health (Beck et al., 2018). Exacerbating this problem is the underrepresentation of racial and ethnic minorities in health science professions, as well as the underrepresentation of women in prominent health research positions (U.S. Department of Health and Human Services, 2017). Because a diverse biomedical, behavioral, and clinical research workforce would be significantly better equipped to serve the nation’s health needs, it is essential for all students to learn about careers in the field of health sciences, which refers to careers and disciplines that focus on improving health through the application of STEM. The health sciences encompass a broad range of occupations and specializations, ranging from clinical work with frequent interaction with patients such as dental hygienists, health education specialists, and nurses and medical doctors to non-clinical healthcare careers such as medical health services managers, medical lab technicians, and biomedical research scientists.

Adolescence offers a key window to promote students’ interest in and self-efficacy—a person’s belief in her or his ability to organize and perform the actions required to reach a desired goal—to pursue health science careers (Ali et al., 2019; Bandura, 1982; Garrison et al., 2021; Glessner et al., 2017; Hernandez et al., 2013; Ozer and Bandura, 1990). As a result of developmental changes, adolescents experience new attractions, motivations, and desires for novel experiences, making adolescence an important time for learning, adaptation, and goal setting (Giovanelli et al., 2020). Applying this concept to health science careers, data on career expectations among eighth grade students strongly predict subsequent areas of study (Christensen et al., 2017); for example, research suggests that those who expected to have a career in science are nearly twice as likely to eventually earn a bachelor’s degree in the life sciences, when compared to students who did not expect to work in the sciences (Maltese and Tai, 2011). Further, data suggests that the gender disparity in interest in science careers present in the eighth grade, and higher rates of science attrition in subsequent years accounts for 69% of the gap in the number of science degrees awarded to male and female college students (Legewie and DiPrete, 2014). Engaging students’ interest in health research careers and health professions in middle school, building competence in fields of study, and keeping students motivated and engaged through high school, is key for building a diverse health sciences workforce.

Health Quest utilizes advances in game-based learning technologies to develop an intervention that enhances adolescent students’ motivation and interest towards pursuing health science careers. Health Quest aims to promote middle school students’ interest in health science careers by: 1) engaging them in a rich, narrative-driven health sciences career adventure game where they interact with virtual characters and explore health science career fields; 2) providing them with captivating videos of health science professions to learn about their career paths; and 3) providing teachers with online professional development materials and in-class support for the implementation of Health Quest in their classrooms. Each of these goals is supported through a specific component of Health Quest: *Health Quest’s game-based learning resources*, which leverage advances in narrative technologies to create engaging health career adventure episodes that introduce students to a broad range of health sciences; *Health Quest’s career discovery resources* which feature video interviews with health professionals; and *Health Quest’s teacher resource center* which provide resources and materials to support middle school teachers’ classroom implementations of Health Quest in their classes.

In the following sections, we describe each of these components. We then describe how the research team adapted the game-based learning resources during COVID-19 to include new narratives and game content about responding to public health outbreaks; and how the team has continued to engage with students, teachers, and counselors during the pandemic to gather critical feedback and focus group data to modify and improve Health Quest. Finally, we describe the technology adaptations implemented to support broad dissemination and outreach that aligns with teachers’ needs in remote learning contexts.

## HEALTH QUEST GAME-BASED LEARNING RESOURCES

Health Quest’s game-based learning resources leverage rich 3D commercial gaming as well as 2D visual novel technologies that engage students in a rich story-driven narrative to allow them to explore and learn more about different health science careers. The game content is designed to expose students to health science careers in public health, nursing, medical lab technician, virology and immunology, and other health science careers.

### Health Quest: Outbreak Investigation Game.

**Figure 1.**
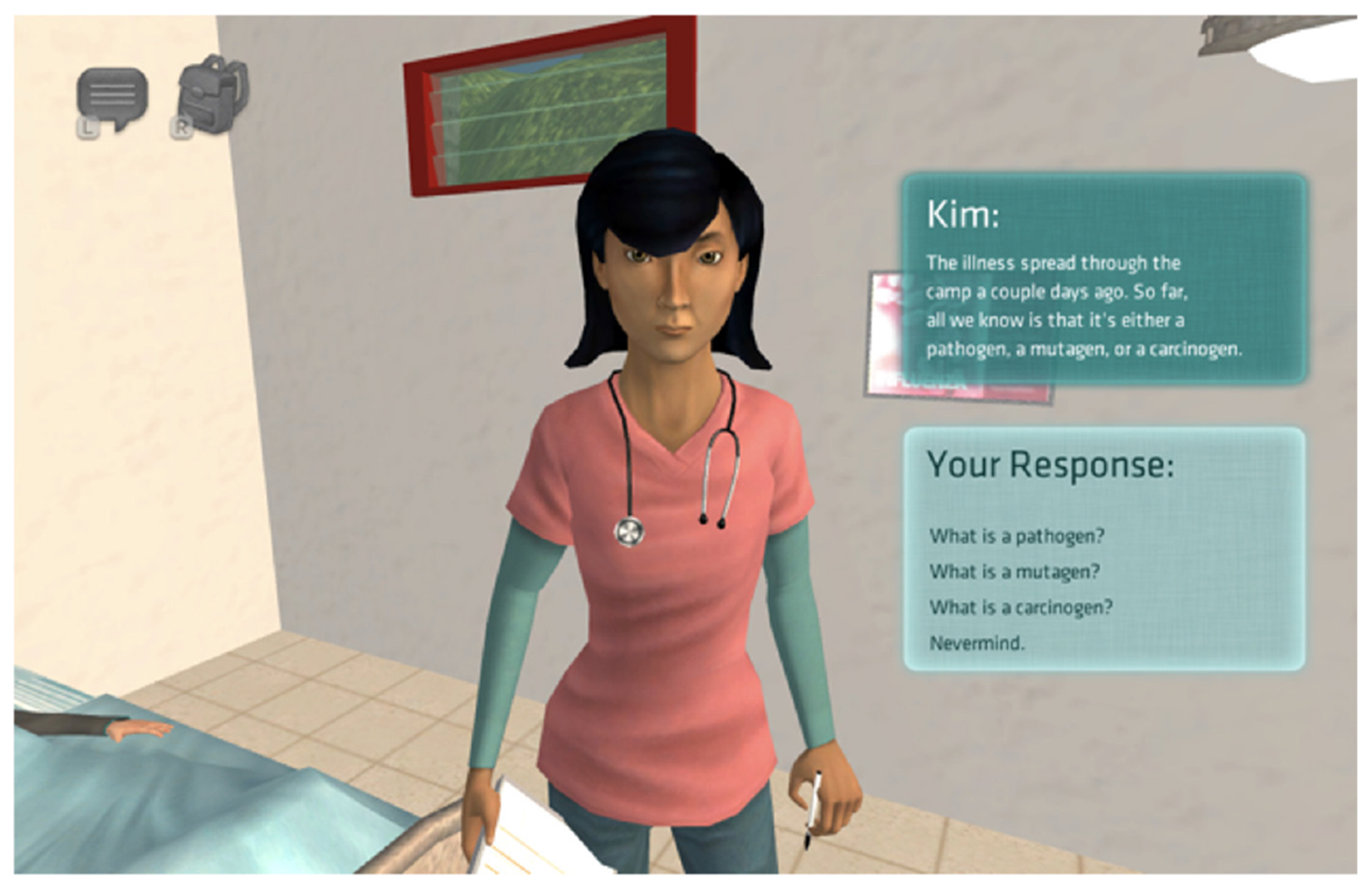
Nurse from Health Quest: Outbreak Investigation game.

Health Quest’s core game-based learning environment, Health Quest: Outbreak Investigation, is a rich 3D immersive science mystery game set on a remote tropical island. The educational content included in the game aligns with the Next Generation Science Standards, the Common Core, and the North Carolina Essential Standards. With an emphasis on microbiology, students learn about pathogens, how they reproduce, and how they spread from person to person while developing an experiential understanding of scientific phenomena. Students play the role of an infectious disease officer and are assigned the task of discovering the identity and source of a disease plaguing the newly established research camp. Students explore the camp from a first-person viewpoint, talk to other virtual camp members, review resources such as virtual posters, diary entries, and virtual objects, and use lab equipment to identify the source of the outbreak (Figure 1). The game features virtual characters who represent various health science fields including a camp nurse, a lead medical lab technician, a lead scientist, a virologist, a microbiologist, a research scientist, and a nutritionist. As students investigate the likely cause of the outbreak, they interact with 3D objects and complete an in-game diagnosis worksheet to record their findings and inferences about the pathogen causing the illness, the disease’s transmission sources, and the proper treatment for the disease. The science mystery is solved when students submit a complete, correct diagnosis and treatment plan to the camp nurse. The game takes players from 60 to 90 minutes to complete.

### Health Quest Career Explorer Episodes.

Upon solving the mystery in Outbreak Investigation, students unlock a series of Health Quest Career Explorer episodes that highlight health science careers associated with characters in Outbreak Investigation, including nursing, medical laboratory technician, and biological sciences. The episodes utilize a 2D visual novel game-style through which players participate in the narrative by reading character dialogue, selecting, and interacting with virtual objects in the environment, and completing activities related to the career field highlighted in the narrative.

**Figure 2.**
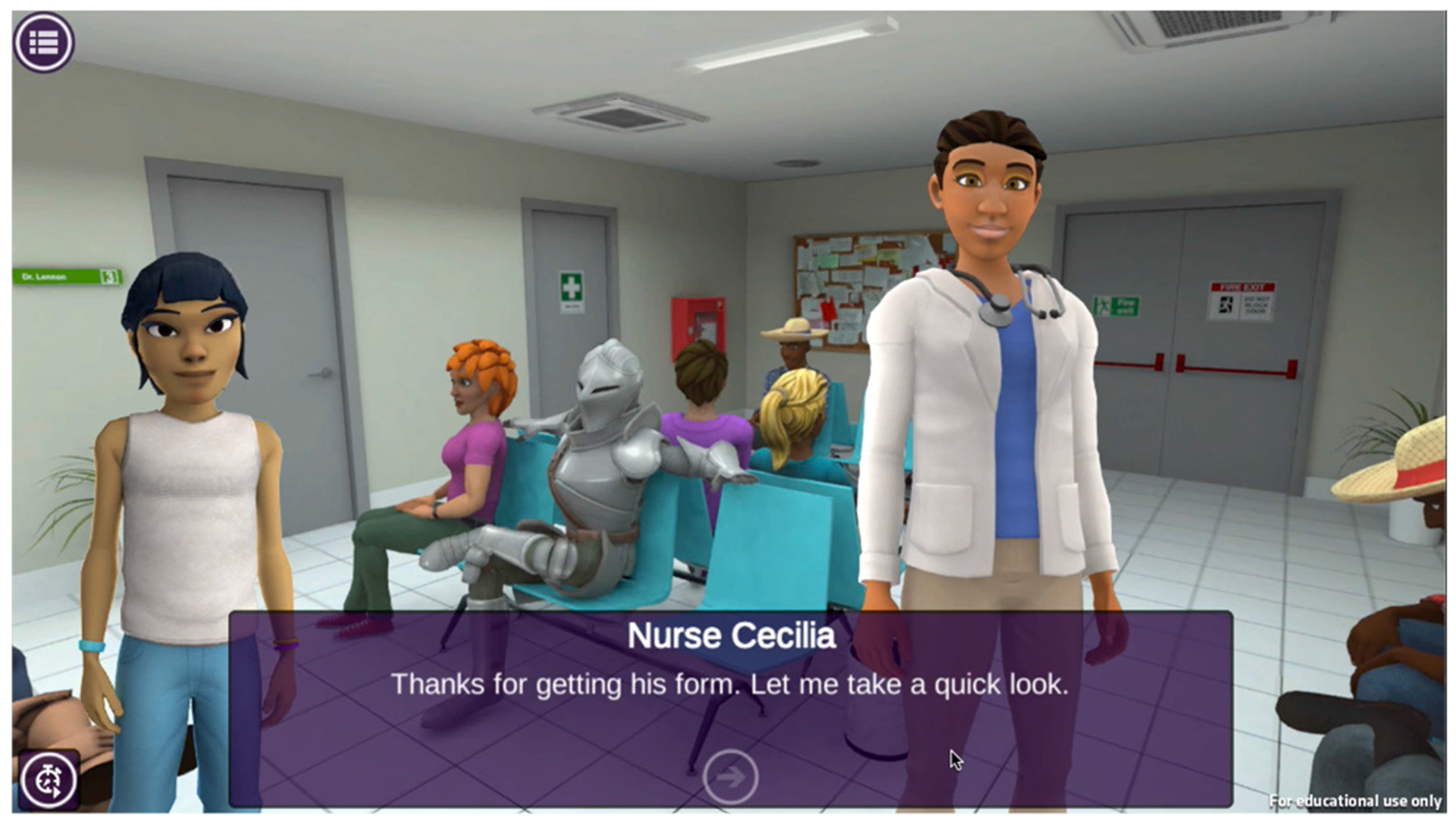
Health Quest’s nursing game episode.

The episode narratives were originally designed to allow students to work collaboratively with virtual characters in the story world to solve challenging research problems for the associated Health Quest career field. For example, in the original Nursing episode, Nurse Kim, the camp nurse from Outbreak Investigation tells a story of a memorable experience she had as an adolescent that led to her career interest in nursing. The narrative takes place in a hospital emergency room and students take on the role of an adolescent-aged Nurse Kim who must help an Emergency Room Nurse deal with a large influx of people from a multi-car accident (Figure 2). Students interact with three other patients with non-urgent concerns in the emergency waiting room with the goal of helping them complete a patient information form. As students interact with the patients, they try to identify injuries and preexisting conditions that might impact each patient’s treatment plan and report this information to the nurse. However, as described below, during COVID-19 the team revised the episode narratives to highlight how professionals in these careers solve challenging health science problems such as preventing and treating outbreaks.

### Health Quest Career Discovery Videos.

**Figure 3.**
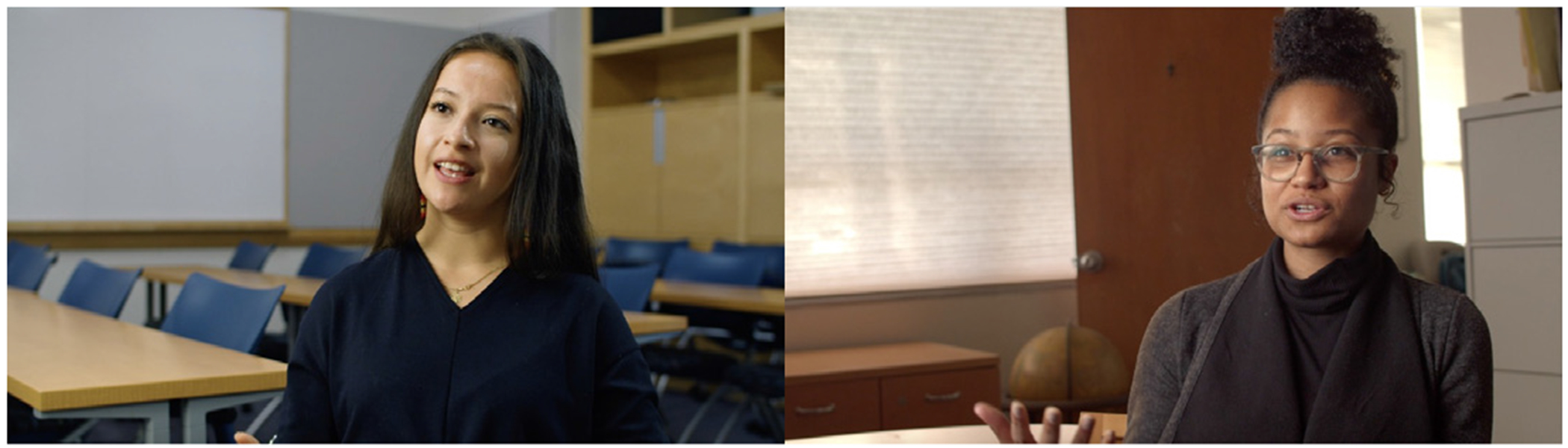
Health Quest’s career interview videos with professionals and near peers.

Another core component of Health Quest is a set of role model interview videos with health professionals. The interview videos feature testimonials from health science professionals in nursing, public health and health disparities research, microbiology, biophysics, medicine, psychology, and medical laboratory sciences. The videos provide accounts of how these professionals became interested in their career fields, how they prepared for their careers, and how personal experiences shaped their career decisions (Figure 3). The role model interviews also include a diverse set of near peers–students and those still training for a profession–who talk about their interests and goals of pursuing a career in the health sciences. Specifically, the videos provide accounts of how near peers have navigated academic and other obstacles as they work towards degrees in health sciences. By including interviews of students who more closely align with middle grade students’ own ages and backgrounds, the set of near peer videos aim to make a stronger impact on middle grade students’ interests and efficacy for pursuing health science careers.

**Figure 4.**
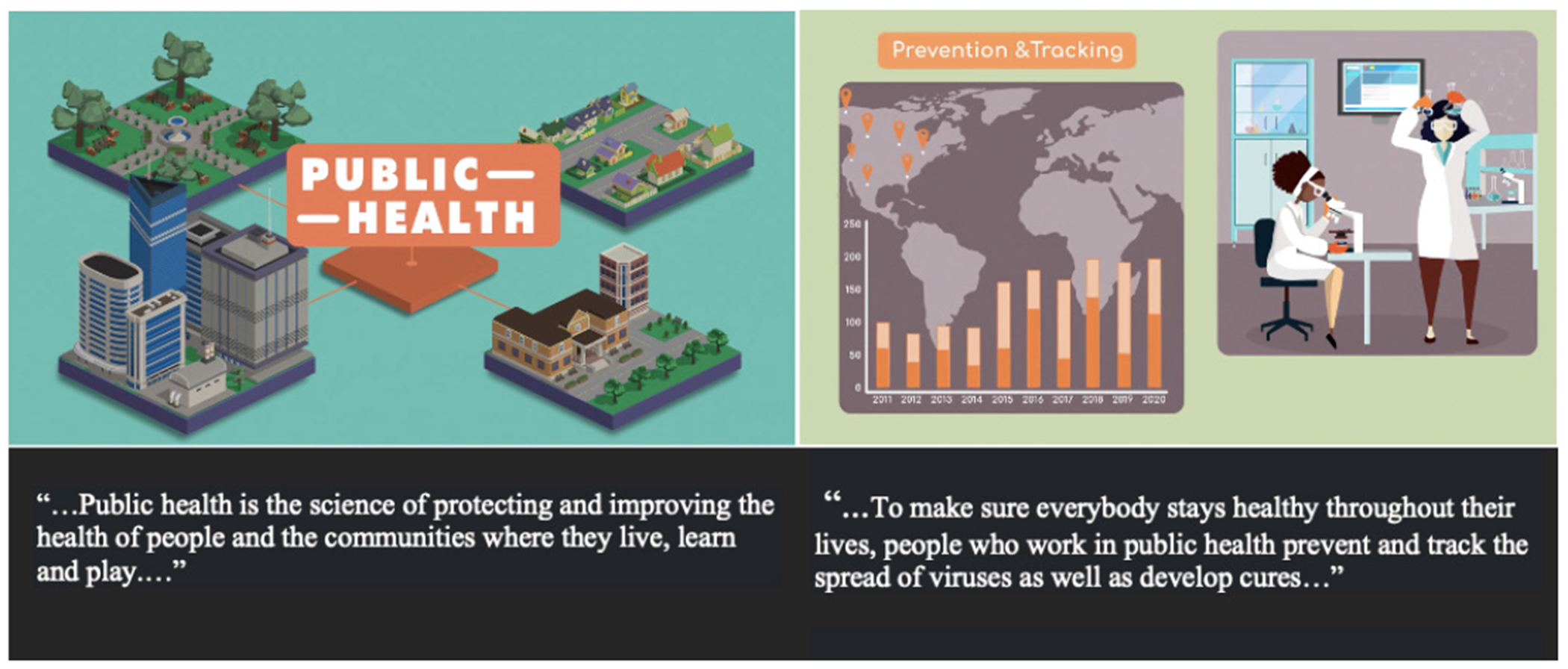
Health Quest’s public health explainer video and accompanying narration that describes how public health professionals use science to investigate and identify public health problems and solutions.

To complement the role model videos, the Health Quest career discovery center also includes a series of animated explainer videos that provide foundational information about specific health science careers. Animated explainers have been used in many fields, including the health sciences, to convey complex scientific phenomena to a wide audience in an easily understood manner. Research shows that animated educational videos can lead to better knowledge retention of concepts compared to traditional lecture-based explanations, particularly for learners with low prior knowledge about a concept, and that students show a strong preference for this educational medium (Bello-Bravo et al., 2018; Fiorella and Mayer, 2016). The current set of Health Quest Explainer videos highlight careers in public health, nursing, medical lab technician, and vaccine research and provide introductory information about each career field, including typical job duties, where these professionals typically work, and basic educational requirements and their role in responding to outbreaks (Figure 4). The videos are two to four minutes long and are designed to provide students with captivating information about the specific career field.

### Health Quest Teacher Resource Center.

In addition to the game and video-based resources, Health Quest includes a Teacher Portal that serves three key roles to help teachers implement Health Quest in their classrooms. First, it provides teachers with an overview of the technical requirements and computing resources needed to play and access Health Quest’s game-based resources. Second, it provides access to a portal where teachers can create and add student accounts, create sign-in credentials, and configure game settings to control which features are active during the implementation. Third, it includes a short overview video of the game experience so teachers can quickly review what students will be doing during game play.

## CHANGES MADE DUE TO COVID-19

The COVID-19 pandemic has impacted many STEM outreach activities and programs and drastically changed educational practices. In response to these challenges, the Health Quest project engaged in several key activities to adapt the game content and the technology underpinning Health Quest’s game-based learning resources. The purpose of these adaptations was to make the game narratives closely connected with addressing public health outbreaks, to support virtual pilot testing, and to ensure students can access and engage in our content remotely, both in formal and informal educational settings. This has included undertaking both a major design initiative, creating a new design at the narrative level, and modifying the gameplay mechanics and assets to support COVID-19 subject matter. It has also included embarking on a substantial reworking of the game environment software to support the launch of a scalable implementation of a COVID-19 version of Health Quest: Outbreak Investigation that can be brought to the general public and support virtual implementations.

### Updating Health Quest’s Game Content.

#### Narrative Updates.

A central component of Health Quest’s game-based learning resources is Outbreak Investigation, an engaging first-person science mystery that introduces students to field work in public health. To make the game’s educational content more germane to the current pandemic, the research team developed new art assets and learning resources, including new posters, textbook entries, and concept matrix items that provide educational information about COVID-19 (Figures 5a and 5b), and began integrating this information into the game. The new in-game posters provide students with summative information about the known symptoms and causes of COVID-19 and the textbook entry provides more detailed information about the causes, transmission vectors, symptoms, and prevention and treatment options for combating COVID-19 and other diseases. Each textbook entry also includes a check on learning activity to ensure students understand the content presented in the passage. When students submit their responses, they receive explicit feedback regarding the accuracy of their answers.

To facilitate the development of these new resources, the research team consulted information from the Centers for Disease Control and Prevention’s website and met with medical experts as well as psychologists and public health experts at the University of California, San Francisco to ensure the content was accurate based on the scientific community’s evolving understanding of the disease.

**Figure 6.**
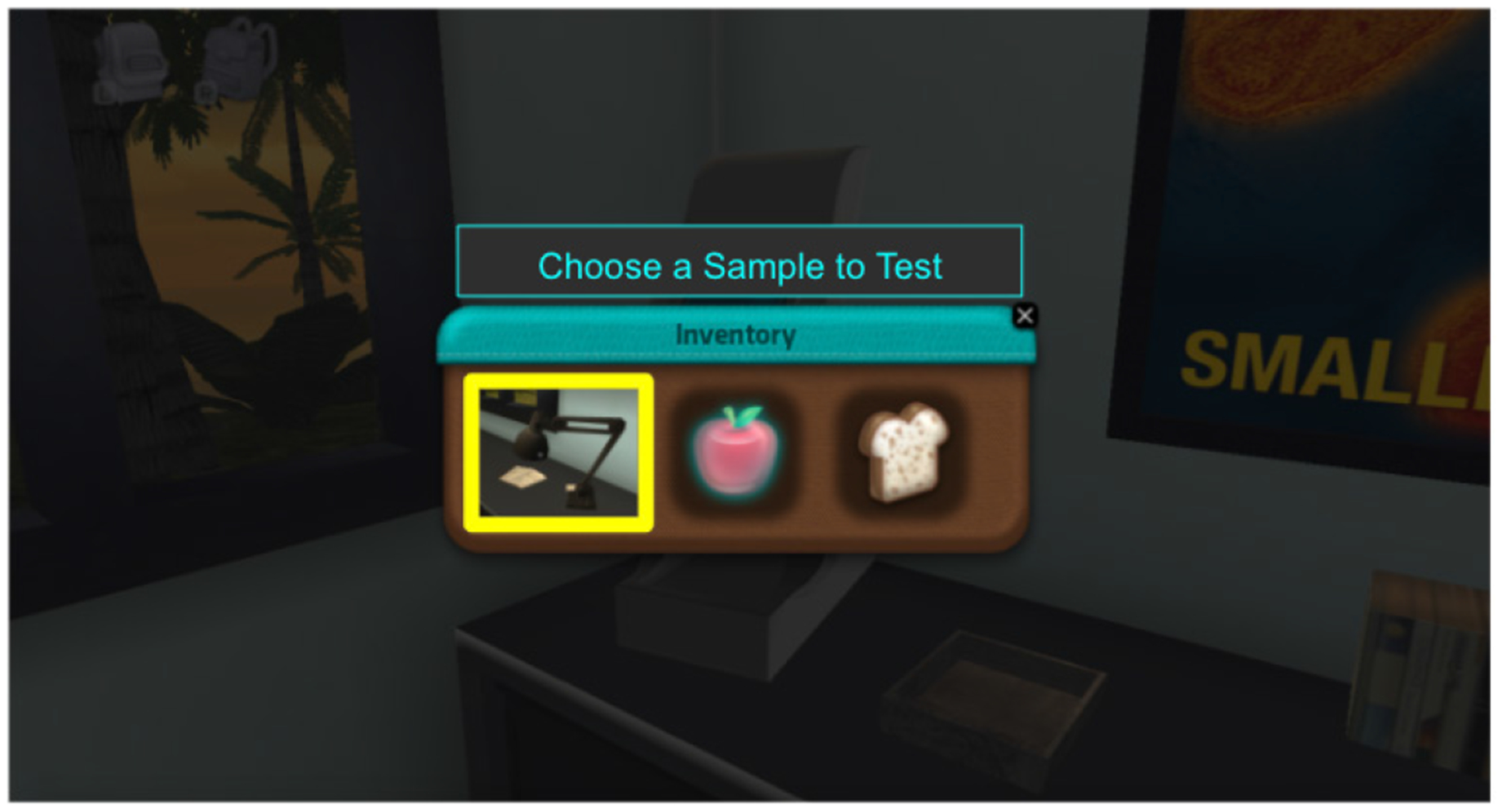
Updated gameplay mechanic.

In addition to incorporating new explorable learning assets in Outbreak Investigation, the research team also made refinements to the game’s core game mechanics and game architecture to enhance players’ gameplay experience and made several technology adaptations to support broad dissemination of Health Quest’s game-based content. In particular, the team refined how players collect and test samples in-game to resemble real-world testing more closely. In the original version of the game-based learning environment, students collected virtual objects and placed them in their inventory bag for testing. For the COVID-19 version of Outbreak Investigation, the game experience was redesigned to enable students to collect samples from objects and surfaces to test these samples for bacteria and viruses using the camp’s lab equipment. The surface samples appear in a player’s inventory bag along with other objects that he or she has collected (Figure 6). In addition to refining this game mechanic, the team updated character dialogue within the game to better differentiate between symptoms of possible illnesses and updated the list of possible symptoms and causes in the in-game diagnosis worksheet to include COVID-19 as a possible cause. Finally, the team has redesigned the game’s tutorial to support the new gameplay mechanics, which has included updating the tutorial instructions and creating new assets and lip-sync mechanics for the virtual game characters.

In parallel with the team’s work to include educational information on COVID-19 in Outbreak Investigation, in the Spring and Fall 2020 we began to refine the original narratives of Health Quest’s career adventure episodes with the goal of highlighting the critical role that professionals in nursing, medical laboratory testing, and vaccine research and immunology play in combating public health outbreaks. Table 1 contains a comparison of the plots from the original episodes to the updated episodes. Refining the episode narratives and plot treatments was an iterative and collaborative process. It involved working with a professional writer who had extensive experience writing narratives and short stories for comics and interactive narrative experiences to update and refine the plot treatments for each episode. As part of the process, the team reviewed media and scientific articles that discussed ways medical professionals were treating COVID-19 cases. We also researched job duties and tasks that could be modeled or included as in-game activities for each career track. Then, we created revised scripts and developed new in-game activities that students would complete. Once these assets were integrated into the game episodes, we completed several rounds of internal testing to gather feedback and critiques of the story narratives, character dialogue, game play instructions, and game play experiences to refine the episodes and in-game activities and content. The updated episode narratives, which are described below, highlight how each health science career field plays a role in solving challenging health problems such as preventing and responding to outbreaks.

#### Medical Laboratory Technician Episode.

**Figure 7.**
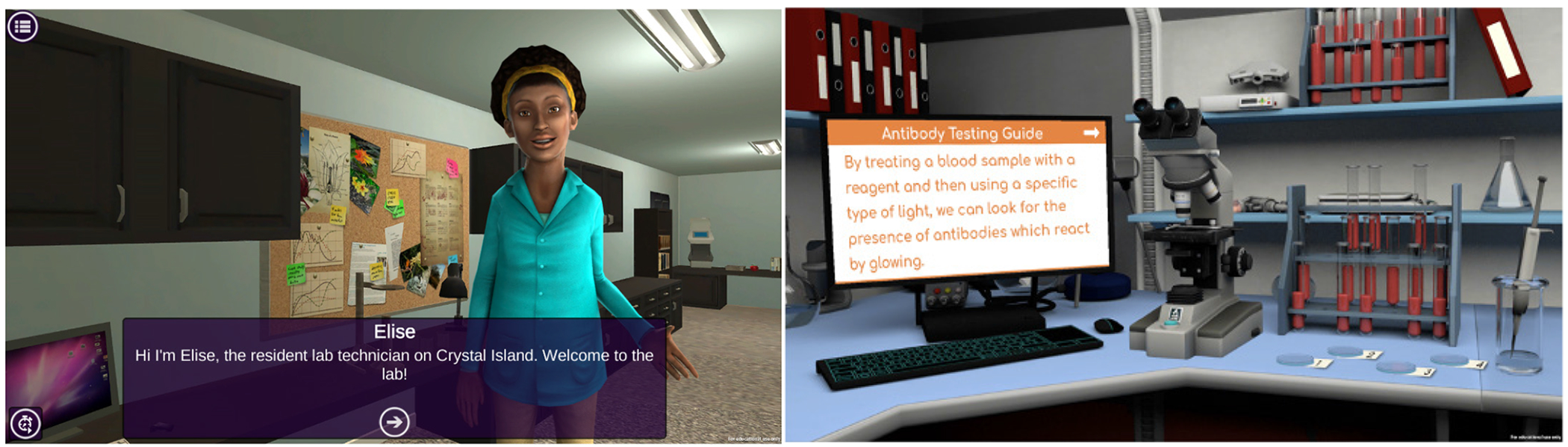
Health Quest’s medical laboratory technician game episode.

The medical laboratory technician episode storyline engages students in a narrative centered around conducting antibody testing and using plasma to treat a novel outbreak (Figure 7). Within the episode, students interact with Elise, the lab technician from Outbreak Investigation to learn about blood, blood types, antibody testing, and how convalescent plasma can be used to treat novel outbreaks. Students gain mastery of these concepts and learn how to use lab equipment to test samples and by completing blood matching and antibody testing games and activities in the episode. Students are also introduced to scientific research that discusses how plasma was used during the early points of the COVID-19 pandemic to treat critically ill patients with the disease.

#### Vaccine Scientist Episode.

**Figure 8.**
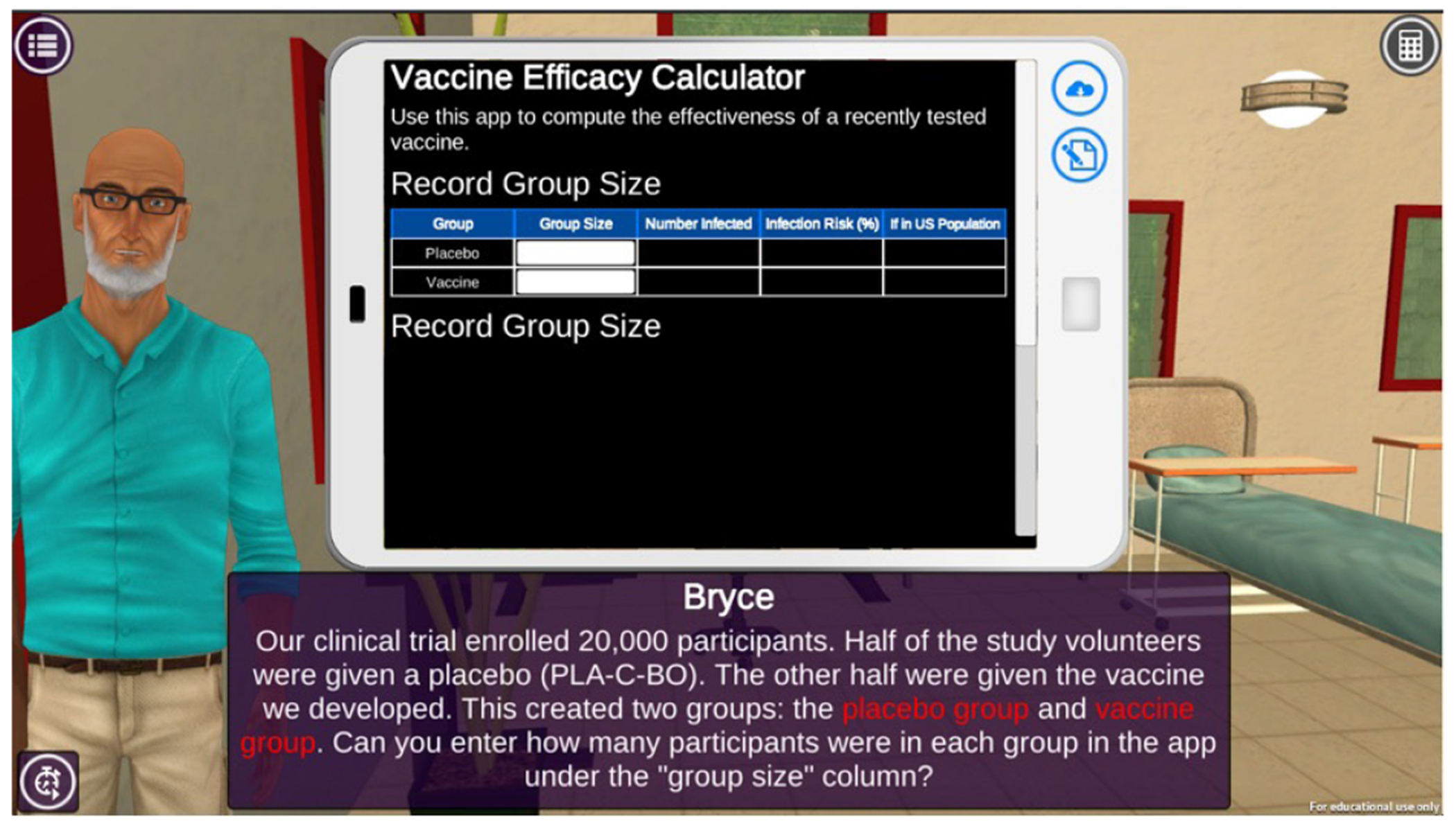
Health Quest’s vaccine scientist game episode.

The vaccine scientist episode engages students in a narrative centered around investigating the efficacy of a newly developed vaccine. Students work with the Lead Scientist from Outbreak Investigation to calculate the vaccine’s efficacy using data collected from a recently concluded clinical trial. Students read a description of the clinical trial study that was conducted, identify the number of participants in the control and treatment groups, calculate the infection risk, extrapolate the numbers to investigate the vaccine’s impact when applied to larger populations, and calculate the vaccine efficacy rate (Figure 8). The activity offers students support in the form of hints and feedback to minimize cognitive load and promote mastery. Similar to the medical lab technician episode, by engaging students in simulations of authentic tasks performed by vaccine scientists, the episode aims to increase students’ beliefs and judgments about their own ability to perform tasks that vaccine scientists complete.

#### Emergency Room Nurse Episode.

The emergency room nurse episode, which is still being refined by the research team, centers on responding to a massive influx of patients at a hospital who each display different symptoms, some of which are linked to a novel outbreak. Students interact and assist the nurse in collecting information from specific patients with the goal of identifying symptoms and preexisting conditions that might impact each patient’s treatment plan.

Students unlock the Health Quest career explorer episodes after completing Outbreak Investigation and each episode appears on the game mission selection screen as a new task that students can complete. The episodes are compatible with WebGL standards, web-based, and playable in a standard web browser.

#### Technology Adaptations.

The COVID-19 pandemic is forecasted to cause an unprecedented impact on academic achievement. Recent projections suggest the average US student will have lost one-third of their expected progress in reading and half of their expected progress in math (Kuhfeld et al., 2020). These declines can be attributed to disparities in computing access, disparities in broadband connectivity, and disparities in learning interactions with teachers. Recognizing these challenges and the need to transition from an in-classroom implementation and dissemination model to a remote and distance learning model, the Health Quest research team has made several technology adaptations to support broad dissemination of Health Quest’s game-based learning resources.

First, the team updated Outbreak Investigation to be playable in a standard web-browser such as Chrome, Edge, and Safari, so that lower powered and less expensive computers such as Chromebooks can access Health Quest’s game-based learning resources. This is in contrast to the original implementation of Outbreak Investigation that was a stand-alone application that required students to download the application to a standard laptop or desktop to play. Porting the game to a web-based application makes Health Quest more accessible to all students regardless of their access to computing resources.

Second, the team upgraded Health Quest’s cloud-based architecture to support large simultaneous deployments to a wide audience base. This update allows hundreds of students to log in and play Health Quest simultaneously, which is in contrast to research-sized data collections in classrooms.

Third, the team integrated the Health Quest Career Adventure Episodes and Outbreak Investigation into the same core game architecture. Game episodes appear as a game activity on a newly designed start screen for Health Quest and students can freely explore the game-based learning resources or, as an alternative implementation, the episodes can be gated so that students must complete activities in a set sequence. Prior to this integration, Outbreak Investigation and the Health Quest Career episodes resided on two different software architectures, and each required different usernames and passwords. Students can now use a single username and password to log in to Health Quest and select among a series of activities to complete, including Outbreak Investigation. Health Quest saves each student’s game data and in-game progress to a cloud-based server, which allows them to exit the game and pick up where they left off. A summary of the changes between the original version of Outbreak Investigation and the updated version is in Table 2.

## FOCUS GROUPS AND PILOT TESTING WITH MIDDLE GRADE STUDENTS DURING COVID-19

Health Quest’s research centers on investigating the impact of Health Quest on middle school students’ knowledge of, interest in, and self-efficacy for pursuing careers in health science fields, particularly for underrepresented groups. The COVID-19 pandemic has not only influenced the team’s design and development decisions, but also impacted the testing and implementation plans for Health Quest. Creating effective and engaging game-based learning resources takes many rounds of focus groups, pilot testing, and feedback from adolescents to ensure game content, narrative, and appearance are tailored to the expectations of our target sample. Over the past year, many of our plans for conducting focus groups have been altered as teachers and students have dealt with the many challenges associated with attending and planning virtual school assignments and balancing student engagement for both on-screen and off-screen activities. Similar to other STEM outreach projects, our team has adapted by planning and conducting virtual focus groups. Because Health Quest’s learning resources are web-based, we have been able to distribute prototype episodes and game content to focus group participants and collect feedback in online video conferencing sessions.

For instance, in Spring 2021, the team conducted a virtual focus group with a small cohort (*n* < 5) of middle grade students and mentors from an after-school program to gather feedback on the design of the Health Quest Medical Laboratory Technician episode. The focus group was conducted remotely using Zoom due to COVID-19 restrictions and lasted approximately one hour. The team worked with the North Carolina State University IRB office to update the focus group protocols and procedures to conduct remote testing and focus groups. As part of the IRB amendment process the team included a summary of how Zoom was going to be used to facilitate the focus groups and students received an incentive for participating. Students completed the Health Quest medical laboratory technician career episode, watched the associated medical laboratory technician role-model video, and watched a short, animated explainer video that discussed medical laboratory technician careers. Then, students engaged in discussion with members of the research team about what they enjoyed and what could be improved. The feedback that the team gathered revealed that the game episode could benefit from having more character animations, more opportunities to interact with characters, and more sound effects. Students also indicated that it would be helpful to include voice-over of the text to make it more immersive, and to reduce the amount of text to read, in general. The team is using this feedback to further refine the narrative and add new gameplay features to increase student agency in the Health Quest episodes.

In addition to conducting focus groups, the project team pilot tested Health Quest in Spring 2021 with several 7th and 8th grade science and health classrooms which included a mix of students who were in classroom and remote learning settings. The purpose of the pilot test was to gather insights on students’ gameplay experiences and to gather teacher feedback on how Health Quest can be incorporated into middle grade science classrooms. A second goal of the pilot test was to test a classroom implementation model that required students to play Outbreak Investigation as a preparatory learning experience prior to playing the Health Quest Career episodes. A guiding research question with implementing this model was how best to divide and sequence the game play experience so that it is feasible to implement in a middle grade science curriculum. Outbreak Investigation takes on average 80 minutes for students to complete, and the Health Quest Career episodes, role model videos, and explainer videos take several class periods to complete as well.

Approximately 120 seventh and eighth grade students were recruited to participate in the pilot test; teachers made the game available to all recruited students and set aside dedicated classroom time for the testing sessions. The pilot test followed a 2-day implementation model. On Day 1 students logged into the game, watched a short 3-minute video that provided a backstory about the outbreak mystery they needed to solve, and played Outbreak Investigation for 30 minutes (student’s game experience timed out when the 30-minute limit was reached). On Day 2, students played one of the available Health Quest career adventure episodes, watched the associated career explainer video, and watched a role model video of a health science professional in the associated career field. While all students were allowed to play the game, parental consent was only gathered from 40 students (45% female) to use their data for research purposes. After the two class periods of game play, the team conducted focus groups with small cohorts of students to gather feedback on their experiences using and playing Health Quest. The team also conducted focus groups with the teachers to gather insights on how to better support the implementation of Health Quest in their classrooms and in remote learning settings.

An analysis of students’ game-trace data groups showed that many students did not complete the 30 minutes of game-play for Outbreak Investigation until Day 2 and that less than half the sample completed the supporting Health Quest career episodes on Day 2. Student and teacher responses gathered from the focus groups suggested that students were deeply engaged in Outbreak Investigation and that more class time should be set aside for gameplay and for completing the Health Quest career explorer activities.

Teacher focus group findings highlighted the need to provide teachers with additional in-class support materials and resources. Specifically, teachers indicated the desire to have an instructor dashboard so they could view students’ in-game progress at multiple levels of granularity and so that they could better address students’ questions about game sequencing and game progress. Teachers also noted they would have benefited from having a better understanding of how students advanced from Outbreak Investigation to the Health Quest career explorer resources. To gauge game progress and engagement for students learning virtually, teachers used individual breakout rooms in Zoom and asked students to share their screens and discuss what activities they had completed. This feedback indicates that in addition to providing teachers with a standard set of instructions for using Health Quest in their classrooms, the Health Quest teacher portal should include a classroom management and dashboard that allows teachers to monitor and track students’ progress.

Students who completed the pilot testing activities in the classroom reported experiencing some lag and long loading times while playing the game, and follow-up conversations with the teacher for this particular group indicated that bandwidth limitations are a common problem in the school. The research team is using these insights to refine the classroom implementation model and develop additional teacher in-classroom support materials.

## DISCUSSION

The COVID-19 pandemic has had a significant impact on STEM teaching and learning, but there has been a robust response from STEM educators to address these challenges by creating, converting, and sharing online resources to meet the needs of students. Leveraging game-based learning technologies, the Health Quest project focuses on developing and disseminating technology-rich resources to broaden the interests of adolescents in biomedical, behavioral, and clinical research careers. The project centers on the development of technology-rich learning resources: A game-based learning environment featuring health careers; video interviews with health professionals from a range of health science fields; and animated videos that provide middle grade students with engaging descriptions of health science career fields. In this article we have described how the project has responded to the challenges presented by the COVID-19 pandemic. We have discussed how the research team is adapting the Health Quest Career Adventure Game to remote learning, including highlighting the role science plays in addressing public health outbreaks. We have also described new gameplay features that have been added to support career modeling and how we have adapted the core technology underpinning Health Quest to support broad dissemination to meet the project’s broader goal of increasing interest and engagement in health science careers. Finally, we discussed how we have used virtual focus groups and pilot testing to gather feedback from adolescents and middle grade teachers to guide the development of Health Quest.

Conducting virtual focus groups and pilot testing during the pandemic has offered tremendous insights for integrating Health Quest in middle grade classrooms. However, our transition from in-person focus groups has not been without challenges. Below, we share some lessons learned from our recent experiences that we plan to use moving forward for our virtual and in-person testing and implementation.

### Recommendation 1.

#### Allocate additional time for testing in a virtual classroom setting.

Virtual pilot testing new learning resources in classrooms requires a significant amount of planning and coordination. It requires working with teachers to identify the optimal amount of classroom time to dedicate to testing, without encroaching on other class requirements. We utilized a 2-day testing model in our pilot test because we anticipated that this would allow the team to gather responses from students on all of the Health Quest activities and that it would allow teachers to fit the game within a small unit for health or science during their normal class day. In retrospect, we should have planned for one additional day of testing to provide a buffer for any unforeseen obstacles that may have impacted our testing plans such as poor internet connectivity which can impact game-play experience and time since the game is web-based. Further, as one teacher in our pilot test indicated, despite providing students with login information prior to the set aside periods for game play, students still took longer than expected to begin the game. Some students encountered technical challenges loading the game via their browser. Moving forward we will work to scale the pilot testing activities to better match the classroom time we have available. We are also continuing to update the game-based resources to optimize them for web-based playing. In cases with extremely low internet bandwidth, we have developed downloadable versions of the game that students and teachers can download and run natively on their laptops and computers.

### Recommendation 2.

#### Provide technical support during virtual pilot testing.

Another challenge of conducting virtual pilot testing compared to pilot testing in person is that project staff are not onsite to help with troubleshooting. To address this, our team has set up virtual technical support rooms using Zoom for teachers and students during the scheduled testing time for our pilot test. This practice has worked great in practice and allows teachers and students to interface with the team and report technical problems via the chat or video if they arise.

### Recommendation 3.

#### Provide teachers with demo accounts to preview and play game-based learning resources.

Allowing teachers to preview the game and game materials is an integral part of providing teachers with the resources they need to support the implementation of Health Quest in their classrooms. To support this ability, we have developed a 2-minute video that teachers can quickly watch that provides an overview of the game and shows what students will be doing in the game. We also provide teachers with a demo account so they can play the game themselves and better understand what students will be asked to do.

In addition to these updates, the team is modifying how students and teachers can sign up and explore the game. Specifically, a new “account sign-up” process is being developed to enable parents to create or verify accounts for minors. These changes aim to support a scalable, publicly available version of Health Quest that will allow students and teachers to easily access the project’s learning content on low-cost devices such as Chromebooks. The team is also working on enhancement to the teacher portal to provide teachers with a pedagogical dashboard that will allow them to review students’ performance on individual activities, and track students’ progress through the game.

## CONCLUSIONS

As teachers and students continue to adapt their teaching and learning practices and activities to respond to the learning challenges resulting from the COVID-19 pandemic, our research team hopes they will be able to utilize Health Quest to engage in authentic and meaningful health science career learning experiences in both remote learning contexts and in classroom-based learning as they shift back to in-person classrooms. Our goal is to introduce middle grade students to the exciting world of health science careers and highlight the many career paths students can take towards becoming a health science professional. Promoting career interests and self-efficacy towards health science careers is critical during adolescence. The Health Quest project aims to meet this need with the ultimate goal of supporting a diverse health sciences workforce. Teachers, students, and researchers who are interested in learning more about Health Quest and about how they can use it in their classrooms and outreach activities are encouraged to visit the Health Quest project website: http://projects.intellimedia.ncsu.edu/healthquest/.

## Figures and Tables

**Figure 5a.**
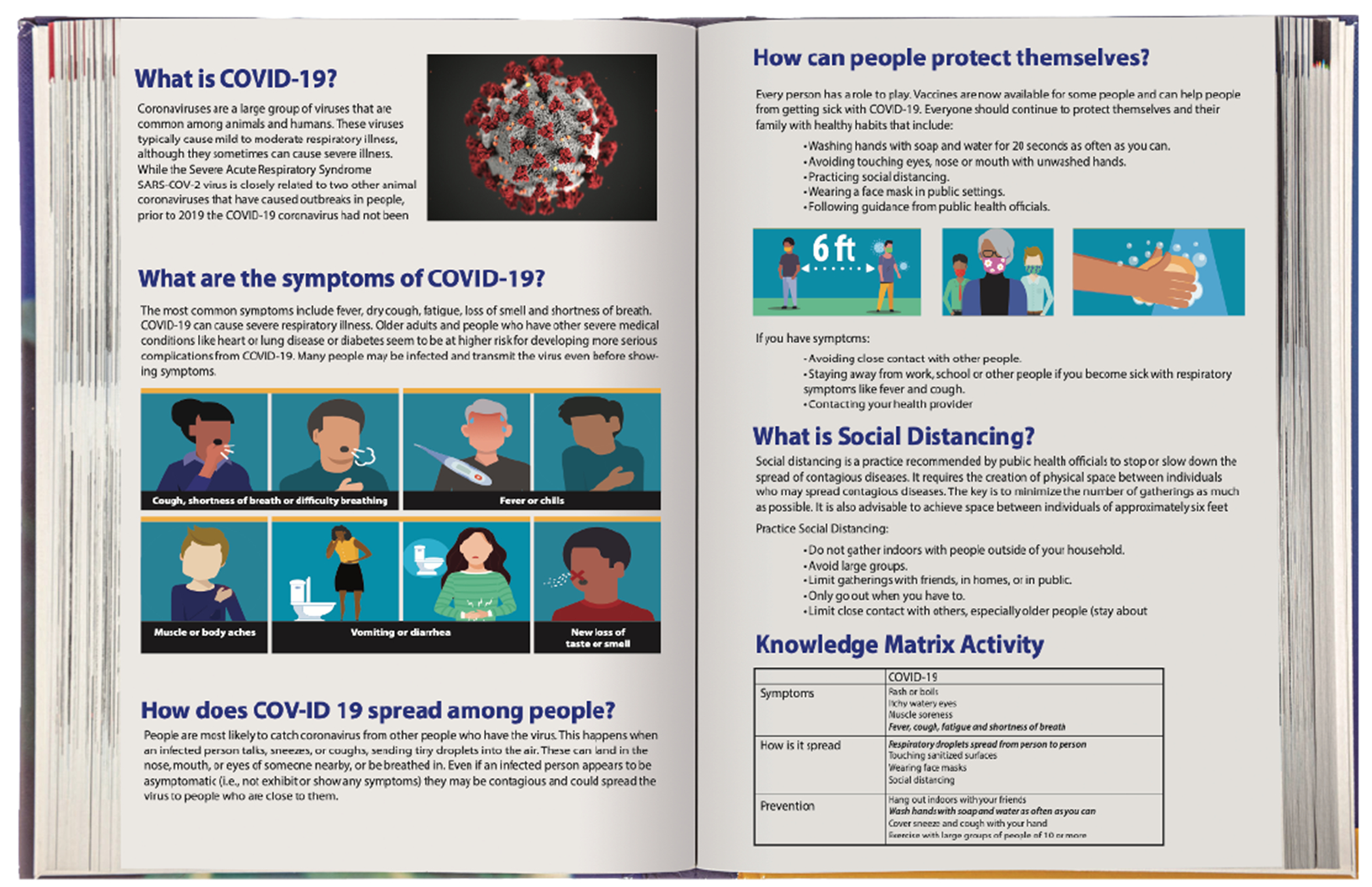
COVID-19 textbook entry in Health Quest: Outbreak Investigation

**Figure 5b.**
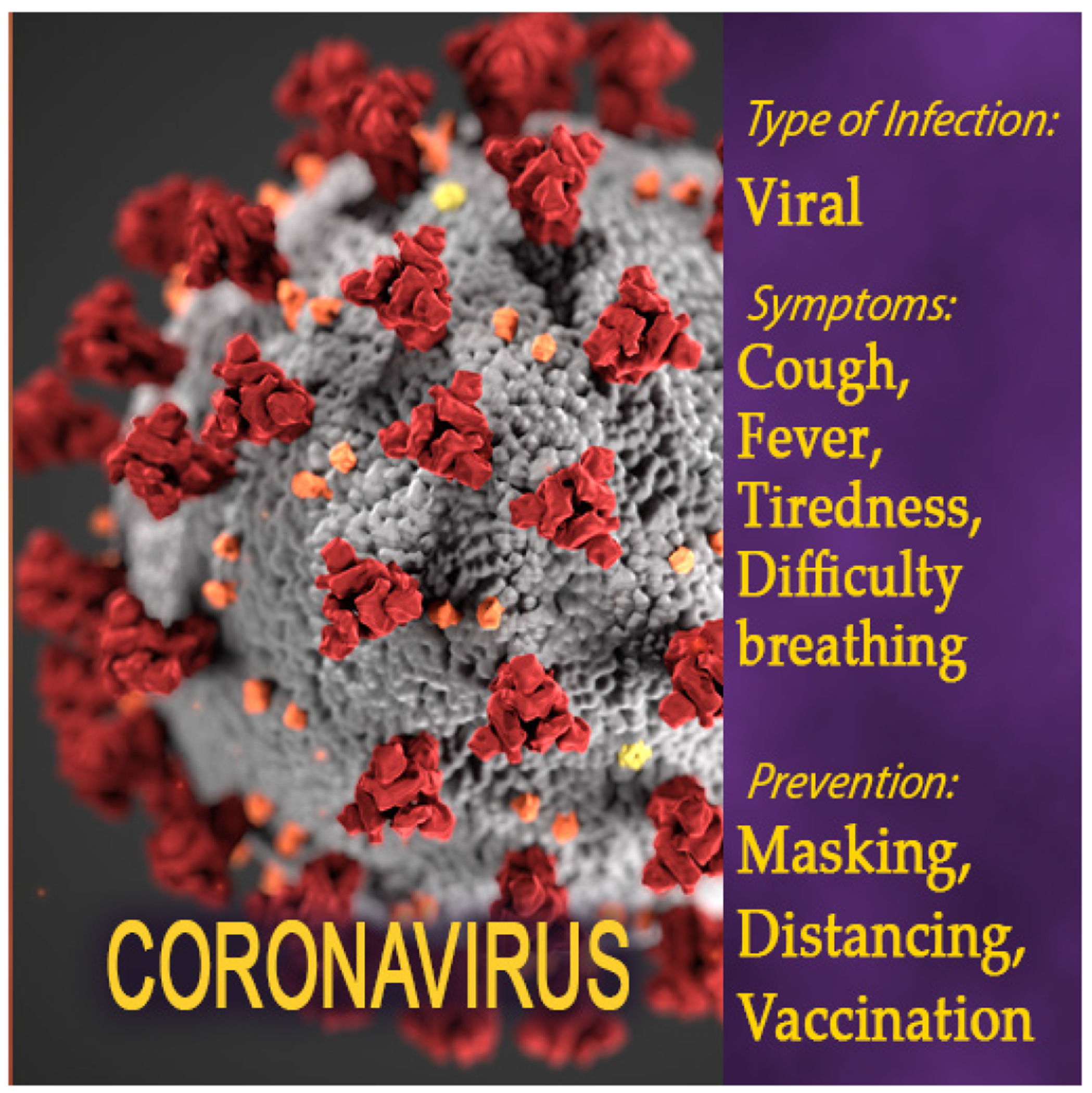
COVID-19 resource poster in Health Quest: Outbreak Investigation.

**Table 1.** Changes to Health Quest’s career explorer episodes during COVID-19.

Health Quest Episode	Original Script	Updated COVID-19 Script
Medical Laboratory Technician	Students learn about and explore telomeres and complete activities to destroy elements that could cause genetic defects/issues. Throughout the activity, students learn what telomeres are, how particular genes cause particular traits, and what research has been able to accomplish in terms of helping cure diseases.	Students work with the medical laboratory technician to help combat the novel outbreak. They learn about blood, blood types, antibody testing, and how convalescent plasma can be used to treat novel outbreaks. Students complete blood matching and antibody testing activities as part of the narrative.
Vaccine Scientist	The original version of this episode was devised to teach students about different aspects of STEM research careers. Students help a scientist complete a series of short activities that focus on the S, T, E, M elements – science, technology, engineering, and math. Students complete activities such as cell counting cells.	Students learn how to test the efficacy of a newly developed vaccine and are introduced to the role that clinical trials play in the process. Students are provided with information about a recently conducted study, identify treatment and control groups, and work with the virtual character to complete a set of calculations to determine the efficacy of a newly developed vaccine.
Nursing	Students interact with three other patients with non-urgent concerns in the emergency waiting room with the goal of helping them complete a patient information form. As students interact with the patients, they complete activities to learn more about each patient’s symptoms and possible causes, and report what they have learned to the nurse.	Students play the role of a nurse and respond to a massive influx of patients at a hospital who display different symptoms, some of which are linked to a novel outbreak. Students interact and collect information from specific patients with the goal of identifying symptoms and preexisting conditions that might impact each patient’s treatment plan.

**Table 2.** Outbreak Investigation technical and content adaptations during COVID-19.

Features	Original Format	COVID-19 Format
Accessibility	Desktop-based application that requires software download and installation; available for Windows PCs and Macs.	Web-based application that is runnable in a web-browser; available for Windows PCs, Macs, and Chromebooks.
COVID -19 content	No COVID-19 learning resources.	Includes game posters, textbook entries, and pamphlets that contain information on COVID-19. Also includes COVID 10 as a possible source in the diagnosis worksheet.
Character dialogue	Utilizes the original character dialogue.	Utilizes revised character dialogue to better differentiate between symptoms of COVID-19 and other viruses.
Game-play mechanics	Students collect virtual objects and test for contagions.	Students collect samples from virtual objects and surfaces and test for contagions.
Tutorial	Includes original instructions for game play controls.	Redesigned tutorial that includes updated instructions for game mechanics and controls.
Deployment size	Limited to classroom-size data collection events.	Cloud-based architecture that supports broad dissemination.

## ABBREVIATIONS

HRSA: Health Resources and Services Administration
MCHB: Maternal and Child Health Bureau
STEM: Science, Technology, Engineering and Mathematics
